# Edible Rose and Roselle: A Critical Review of Nutritional Composition, Safety, and Regulatory Considerations for Product Development

**DOI:** 10.1111/1750-3841.71524

**Published:** 2026-10-19

**Authors:** Temilola Dada, Susanne Neugart, Layla Engelhardt

**Affiliations:** ^1^ Department of Crop Science Division of Quality and Sensory of Plant Products Georg‐August‐University Göttingen Göttingen Germany

**Keywords:** allergenicity, food safety, functional foods, phenolic compounds, product development, regulatory standardization

## Abstract

The growing demand for sustainable and health‐promoting products has increased scientific and industrial interest in edible flowers as functional ingredients. Rose (*Rosa* spp.) and roselle (*Hibiscus sabdariffa* L.) have gained attention for their traditional use, diverse phytochemical composition, and emerging applications in functional foods, nutraceuticals, and cosmetics. Much existing literature treats these species descriptively, reporting compositional data without critically addressing the safety, regulatory, and standardization barriers constraining industrial adoption. This review evaluates their nutritional composition, phytochemical profiles, and techno‐functional properties, while critically addressing cultivar‐level compositional variability, food safety (natural toxic compounds, microbial risk, pesticide residues), allergenicity, extraction methodology standardization, regional regulatory frameworks, and post‐harvest industrial standardization. Rose petals are characterized by volatile terpenoids, flavonol glycosides, and phenolic acids linked to sensory quality and antioxidant activity; documented safety concerns centre on pesticide residue risk from dual ornamental/food‐crop cultivation and oil adulteration. Roselle calyces are rich in anthocyanins, organic acids, polysaccharides, and minerals, supporting use as natural colorants, acidulants, and stabilizers, though cyanogenic glycoside variability, microbiological surveillance gaps, and an almost complete absence of allergenicity data warrant attention. Roselle seed protein, proposed as a functional ingredient, lacks allergenicity characterization. Applications in beverages, bakery products, gels, and cosmetics demonstrate commercial potential, but broader adoption remains limited by compositional variability, bioactive instability, and a persistent gap between demonstrated technical feasibility and adopted, food‐relevant standards for extraction, quality control, and regulatory harmonization. Future research should prioritize dose‐specific toxicological and allergenicity data, validated extraction protocols, and deliberate prioritization of edible flowers within regulatory and standardization mechanisms.

## 1 Introduction

The growing demand for sustainable, health‐oriented, and minimally processed foods has intensified scientific and industrial interest in edible flowers as sources of bioactive compounds and functional ingredients. Among the most commercially relevant plant taxa are rose (*Rosa* spp.) and roselle (*Hibiscus sabdariffa* L.), two botanically distinct plants that have historically occupied important roles in traditional medicine, gastronomy, and cosmetic formulations across Asia, the Middle East, Africa, Europe, the Americas, an d Australia. In recent years, both plant taxa have attracted increasing attention within the functional food, nutraceutical, and natural colorant sectors due to their high concentrations of phenolic compounds, organic acids, pigments, volatile constituents, and structurally diverse polysaccharides (Mulík and Ozuna 2020). Their expanding market relevance is reflected in projected commercial growth through 2033–2034 (Market Data Forecast 2025a, 2025b), which could be beneficial to the beverage, wellness, and clean‐label ingredient industries. Despite this commercial momentum, the industrial integration of rose and roselle remains constrained by substantial variability in chemical composition, sensory quality, and technological functionality. A major limitation in the current literature is the tendency to evaluate these plants either from a purely ethnobotanical perspective or through isolated phytochemical analyses, without integrating species‐specific compositional differences into a broader framework of industrial applicability. Consequently, critical relationships between genotype, environmental conditions, tissue specificity, processing technology, and bioactive stability remain insufficiently synthesized. This gap is particularly important because the nutritional and functional value of rose (*Rosa* spp.) and roselle (*H. sabdariffa*) is highly dependent on cultivar, developmental stage, extraction method, and post‐harvest treatment (Ahmed et al. 2024; Gupta et al. 2023; Zhao et al. 2025). For example, varietal differences in roselle can result in 2–3‐fold variation in anthocyanin content, with cyanidin‐3‐*O*‐glucoside levels reaching up to 446 mg/100 g DM (Mulyaningsih et al. 2025). Furthermore, although rose and roselle are frequently grouped together as edible flowers or botanical ingredients, they differ considerably in their biochemical composition and technological functionality. These differences between rose and roselle influence their suitability for various applications.

The aim of this review is to provide a clearer comparative understanding of their nutritional composition, phytochemical profiles, and functional properties is therefore needed to support standardized and evidence‐based industrial applications. This requires not only technical and functional characterization, but also consideration of compositional variability, food safety and allergenicity, standardized extraction protocols, regulatory requirements, and post‐harvest quality control. Addressing these aspects will be essential to translate the considerable technical potential identified in this and previous reviews into safe and scalable industrial applications.

## 2 Nutritional Composition and Functional Characteristics of Rose and Roselle

Available compositional data for *Rosa* spp. and *H. sabdariffa* remain highly heterogeneous, reflecting differences in genotype and cultivar, cultivation environment, developmental stage, plant tissue, post‐harvest processing, and analytical methodology (Baydar et al. 2016; Cid‐Ortega and Guerrero‐Beltrán 2015; Mallick et al. 2024). This means that compositional values reported across different studies are frequently not directly comparable. This has concrete consequences for product development. Red and pink rose cultivars generally contain higher anthocyanin concentrations and are therefore better suited to applications where pigmentation is desired, whereas pale or yellow cultivars dominated by flavonols are preferable where color contribution should be limited; Section 2.7 (Wan et al. 2019). A specification based on genus or species alone, without cultivar, therefore cannot serve both purposes: it conceals compositional differences that are directly relevant to formulation outcome, not merely to reported nutrient values.

The same applies to roselle, where anthocyanin concentrations vary two‐ to three‐fold across cultivars, with cyanidin‐3‐*O*‐glucoside reaching up to 446 mg/100 g DM in selected material (Mulyaningsih et al. 2025). This variation matters for color intensity, bioactive content, and processing behavior. Roselle anthocyanins lose up to 56% of their content after 60 min at 80°C (Suzery et al. 2020), compared with 18%–38% loss for sour cherry juice anthocyanins over 4 h at a similar temperature range; Section 4.1 (Szalóki‐Dorkó et al. 2015). While these data provide a useful reference for assessing anthocyanin thermal stability, their applicability to roselle products is limited by system‐specific differences in anthocyanin composition, matrix characteristics, pH, and oxygen exposure. Consequently, thermal stability should be determined experimentally for the specific roselle product instead of being extrapolated from other anthocyanin‐rich food systems. Variability also extends to safety‐relevant compounds. Cyanogenic glycoside content differs measurably between red and green roselle cultivars; Section 3.1 (Adanlawo and Ajibade 2006). Available data suggest typical dietary exposure is unlikely to pose acute cyanide risk (Ali et al. 2005). The actual risk depends on concentration, serving size, and exposure frequency parameters that are rarely reported together. The same principle extends to other antinutritional and bioactive compounds whose concentrations vary by tissue and cultivar.

Quantitative variation alone is insufficient for robust ingredient characterization. Meaningful comparison and standardization require botanical identity, cultivar, plant tissue, processing history, and analytical methodology to be considered jointly. Apparent inconsistencies in the literature may therefore reflect differences in source material or analytical conditions, highlighting the need to account for these factors when translating phytochemical data into nutritional, technological, and safety specifications.

### 2.1 Carbohydrates and Structural Polysaccharides

Carbohydrates dominate the macronutrient fraction in both rose petals and roselle calyces, reaching approximately 60%–86% DM in *Rosa* spp. and around 60% DM in roselle calyces (Hegde et al. 2022; Kholaf 2024). This shared magnitude, however, disguises a functional divergence that matters more than the raw percentage; both *Rosa* spp. and *H. sabdariffa* derive their carbohydrate value from fundamentally different fractions, with correspondingly different industrial implications.

In rose, carbohydrate functionality is tied to soluble sugars and their metabolic role instead of structural function. In *Rosa damascena*, sucrose peaks (6.02 mg/g FM) during intermediate flower development before declining sharply at senescence, while glucose and fructose rise during early opening and fall as respiration intensifies (Önder et al. 2023). Because carbohydrate metabolism supplies precursors for terpene and phenylpropanoid biosynthesis, harvest timing becomes a direct lever on aroma intensity, not just sweetness, meaning that a fixed harvest schedule optimized for yield may be actively working against sensory quality. The same fermentable sugars also serve as microbial substrates in fermented products such as wine, directly shaping final aromatic profile (Maicas 2021; Önder et al. 2023), which means fermentation‐based rose products inherit this timing sensitivity as a process variable, not merely a raw‐material characteristic.

The nutritional value of carbohydrates in roselle is mainly related to other components: structural polysaccharides pectins, hemicellulose, cellulose, and mucilage‐like gums reaching up to 78.2% (Jabr and Al‐Mudhaffar 2025) and drive viscosity, gelation, and water‐holding capacity instead of sweetness. This difference is important because rose and roselle cannot be used as interchangeable carbohydrate sources, even if their total carbohydrate contents are similar.

Treating them as equivalent could therefore lead to errors in product development as well as misleading nutritional conclusions. Rose carbohydrates should be evaluated for their sensory and fermentation‐relevant properties; roselle carbohydrates for their property as a functional hydrocolloid. These properties suggest that roselle may have potential as a clean‐label stabilizing ingredient with functional characteristics comparable to hydrocolloids such as pectin or guar gum. In contrast, the carbohydrate fraction of rose appears to have limited structural functionality and is more relevant to processes related to aroma formation and fermentation.

### 2.2 Protein Composition and Functional Potential

Reported protein values for rose petals illustrate the variability problem established above but do not resolve it. *Rosa* spp. contain crude protein in the range of approximately 12%–14% DM (Liu et al. 2024), while a report of 7.6 g/100 g DM is a near two‐fold discrepancy that likely reflects cultivar and methodological differences rather than biological inconsistency, reinforcing why protein content alone cannot be cited without specifying source material and method (Pires et al. 2017). Regardless of the reported concentration, protein in rose petals appears to make only a minor contribution to techno‐functional properties in food systems. Its biological relevance is more closely associated with enzymatic processes and secondary metabolism during flower development. Roselle calyces are comparably low in protein, 4.1%–5.7% DM (Balarabe 2019; Salami and Afolayan 2021), but their nutritional relevance rests on amino acid balance instead of quantity, providing essential amino acids in proportions close to recommended adult dietary allowances (Hoffer 2016).

The most industrially relevant protein fraction in roselle is found in the seeds, where protein concentrations of up to 33.45% DM have been reported (Hainida et al. 2008), approaching levels seen in conventional legumes such as lentil and cowpea (Gerrano et al. 2019; Tahir et al. 2011), and positioning roselle seed protein as a non‐trivial alternative, instead a marginal byproduct. The amino acid profile includes nutritionally relevant essential amino acids leucine (9.9%), valine (3.6%), and phenylalanine (3.2%), supporting fortification use (Mahmoud et al. 2024; Sudik David 2024), and a roasted‐seed beverage formulation has independently reached a comparable protein content of approximately 31.5% DM (Mawouma et al. 2024), indicating that these findings are not confined to a single study and are unlikely to represent a study‐specific artifact. What distinguishes roselle seed protein from a merely adequate nutritional source is its techno‐functional behavior: isolated fractions exhibit emulsifying capacity (95–18 mL/g protein) and oil‐holding capacity (3.47–7.23 mL/g protein) alongside water‐binding ability (Tounkara et al. 2013), and enzymatic hydrolysates achieve high nitrogen solubility (up to 94.17% at pH 12) with favorable emulsifying and water‐holding properties (Tounkara et al. 2014). These are precisely the functional properties that determine whether a plant protein can compete in formulated food systems instead of merely serving as a nutritional additive, which is what makes roselle seed protein a credible candidate for protein‐enriched beverages, bakery systems, and emulsion‐based products instead of a niche ingredient. This functional promise, however, is not yet matched by safety characterization: as established in Section 3.4, no allergenicity data exist for roselle seed protein despite its proposed use in exactly these fortified formats a gap that matters more here than for rose, where protein remains functionally negligible regardless of its safety profile.

### 2.3 Lipid/Oil Composition and Aroma Interaction

Both plants contain low total lipid concentrations 1.30–3.28 g/100 g DM in *Rosa* spp. and approximately 0.9–3.90 g/100 g DM for roselle (Guimarães et al. 2010; M'be et al. 2023; Yashaswini 2011), but this similarity in quantity conceals a sharp functional divergence, since the two plants' lipid fractions serve entirely different technological roles.

In rose, the lipid‐relevant fraction is the essential oil, lipophilic monoterpene alcohols (citronellol, geraniol, nerol), long‐chain aliphatic hydrocarbons, phenylethyl alcohol, and minor fatty acid esters and waxes (Baydar and Baydar 2005; Verma et al. 2011). These compounds are primarily volatile aroma constituents instead of structural lipids, with their functional relevance linked to flavor delivery and potential antimicrobial and antioxidant activities. In probiotic fermented whey beverages, low concentrations of rose oil enhanced both probiotic viability and sensory quality during storage (Dinçoğlu and Rugji 2021). In low‐fat food systems, effective aroma retention may therefore require suitable carrier or encapsulation systems (Guichard 2002; Riéra et al. 2006; Weng et al. 2026).

Roselle seeds, by contrast, contain a fixed‐oil fraction 11%–21% DM (Petraru et al. 2021; Salami and Afolayan 2021), positioning roselle below major oilseeds like sunflower (65.42%) or soybean (19.04%–21.99%) (Fayyaz et al. 2025) but above common vegetables (8.5% fat), and therefore a modest but non‐trivial oil source instead of being discarded as a byproduct (Petraru et al. 2021). This oil is rich in linoleic (36%–39%) and oleic (27%–29%) acids (Naeem et al. 2019; Zarringhalami and Ganjloo 2018) and contains substantial concentrations of tocopherols (approx. 2000 mg/kg), dominated by γ‐tocopherol (74.5%), with smaller α‐ and δ‐tocopherol fractions (Mohamed et al. 2007). The high polyunsaturated fatty acid content of roselle seed oil increases its susceptibility to oxidation, while its relatively high γ‐tocopherol content may counteract this susceptibility. In mayonnaise formulations, the inclusion of roselle seed oil was associated with enhanced oxidative stability, which was attributed to its endogenous tocopherol content despite its high polyunsaturated fatty acid proportion. (El‐Deab and Ghamry 2017; Mohamed et al. 2007). The oxidative stability of roselle seed oil is therefore determined by the balance between its high degree of unsaturation and its endogenous antioxidant content, instead of by its fatty acid profile alone. However, oxidation may still occur under prolonged exposure to oxygen, light, or heat. Thus, the endogenous tocopherol content represents a favorable property for formulation, but does not ensure long‐term oxidative stability.

As in rose‐based systems, lipids may contribute to aroma retention in roselle formulations (Guichard 2002; Riéra et al. 2006). Accordingly, low‐fat formulations may require suitable encapsulation or delivery systems to maintain aroma stability (Madene et al. 2006; Weng et al. 2026), representing a shared technological consideration despite differences in the lipid composition and functionality of the two taxa. Future studies should therefore evaluate lipid functionality, oxidative stability, and antioxidant protection under relevant processing conditions, including their effects within complex food matrices. The compiled macronutrient, energy, and sugar profiles of rose and roselle are summarized in Tables S1 and S2, respectively.

### 2.4 Phytochemicals and Functional Potential

The sensory properties, physiological activity, and industrial functionality of rose and roselle are determined substantially by phenolic acids, flavonoids, anthocyanins, tannins, and volatile metabolites (Table S3), whose accumulation depends on genetic background and growing conditions (Sharma et al. 2019). This intrinsic variability is further influenced by extraction and processing conditions, which affect both compound recovery and the formation of degradation products. Consequently, reported phytochemical concentrations may reflect methodological differences as well as variation in the plant material itself. This matters directly for the values presented throughout this section: a total phenolic or anthocyanin figure reported without its extraction conditions is not yet comparable across studies, let alone across species.

#### 2.4.1 Extraction and Recovery of Bioactive Compounds

Extraction strongly influences the recovery and stability of phenolics and anthocyanins from rose and roselle. Conventional techniques such as maceration and Soxhlet remain widely used because of their simplicity and low equipment requirements, despite longer extraction times and potential thermal degradation (Cendrowski et al. 2017; De Castro and Priego‐Capote 2010).

In roselle, ultrasound‐assisted extraction produced higher anthocyanin and phenolic contents than Soxhlet extraction (Ahmed et al. 2024), while extraction combining ultrasound and microwave energy reduced extraction time and altered terpene recovery from *R. damascena* petals (Patrascu and Radoiu 2016). Natural deep eutectic solvents have likewise increased phenolic and anthocyanin extraction from roselle and may improve pigment stability (Alañón et al. 2020; Zannou et al. 2020).

However, available studies remain highly protocol‐specific and rarely address scalability, cross‐cultivar reproducibility, sensory effects, or food safety. Extraction optimization may also conflict with compound stability; for example, elevated temperatures up to 80°C can maximize roselle anthocyanin recovery during ultrasound‐assisted extraction (Ahmed et al. 2024), yet the same temperature accelerates anthocyanin degradation by up to 56% within 60 min (Suzery et al. 2020). Thus, the extraction method should be considered an important source of compositional variability, and standardized protocols validated across cultivars and production scales are still needed.

#### 2.4.2 Phenolic Compounds and Antioxidant Functionality

Phenolic compounds are among the most industrially relevant bioactive fractions in *Rosa* spp. and *H. sabdariffa* due to their antioxidant, antimicrobial, anti‐inflammatory, and potential antidiabetic properties (Hegde et al. 2022; Rodríguez‐Negrete et al. 2024). However, the two plants differ substantially in both concentration and phenolic identity, a divergence with functional consequences beyond magnitude alone. Reported total phenolic content varies with genotype and extraction method. Values around 212 mg GAE/g have been reported for *R. damascena*, while roselle calyx generally showed lower levels but is rich in hydroxycinnamic acids such as protocatechuic and chlorogenic acid, which contribute reducing capacity and metal‐chelating activity in aqueous systems (Osman et al. 2014; Riaz et al. 2020; Samadi and Raouf Fard 2020; Trendafilova et al. 2023). Treating single‐point TPC values as fixed species constants risks overstating precision, given the broad ranges documented across studies and matrices. Antioxidant activity in both species correlates with anthocyanin and phenolic content but is not solely concentration‐dependent; molecular structure, glycosylation patterns, and synergistic interactions among anthocyanins, flavonols, phenolic acids, and organic acids modulate net performance in complex matrices (Ahmed et al. 2024; Cai et al. 2024; Hegde et al. 2022; Jamrozik et al. 2022). Therefore, TPC alone does not reliably predict functional value, yet this limitation is rarely acknowledged in comparative tables. Practical application is further constrained by sensitivity to genotype, extraction conditions, solvent type, pH, thermal processing, light, and oxidative stress, meaning laboratory‐measured phenolic content should not be assumed equivalent to functional performance in finished, processed products (Dangles and Fenger 2018; Oancea 2021; Yagi et al. 2023).

#### 2.4.3 Anthocyanins: Pigmentation, Stability, and Technological Challenges

Anthocyanins are the most industrially consequential phytochemical class in both species, but their functional weight differs substantially; in rose, anthocyanins (cyanidin and peonidin derivatives) contribute to visual quality in selected red cultivars without serving as a dominant functional ingredient class (Kumari et al. 2022), while in roselle, anthocyanins predominantly delphinidin‐3‐sambubioside and cyanidin‐3‐sambubioside (Li et al. 2024; Salami and Afolayan 2021) are the primary driver of both color and antioxidant value, making roselle a natural colorants candidate in a way rose is not.

This functional value is constrained by chemical instability that is structural, not incidental. Below pH 2, anthocyanins exist predominantly as the stable, red flavylium cation; as pH rises, hydration and structural rearrangement generate colorless carbinol and chalcone forms, degrading both color and antioxidant activity (Castañeda‐Ovando et al. 2009; Enaru et al. 2021). Thermal processing accelerates the same underlying hydrolysis and glycosidic cleavage pathway (Moldovan and David 2014; Oancea 2021; Patras et al. 2010). This is the same degradation route already quantified in Section 2 introduction, where roselle anthocyanins lost up to 56% of content within 60 min at 80°C (Suzery et al. 2020). Roselle beverages are especially exposed, as aqueous extraction removes anthocyanins from their protective plant matrix, and pasteurization, boiling, and infusion each reduce anthocyanin content and shelf‐life stability in practice (Moldovan and David 2014; Wu et al. 2018).

Current stabilization strategies include co‐pigmentation (Pangestu et al. 2020; Wu et al. 2018), encapsulation (Lema et al. 2021), and polysaccharide‐based delivery (Rigolon et al. 2024). Each demonstrates that anthocyanin degradation can be mitigated. However, each targets only one stress factor (pH, heat, or matrix exposure), and none has yet been validated as a combined, all‐in‐one solution. Future work on rose‐ and roselle‐based products therefore needs to treat anthocyanin stability as a multi‐factor engineering problem, with extraction yield, molecular stability, and food‐matrix compatibility addressed jointly instead of optimizing extraction yield and stability separately, as the current literature largely does.

### 2.5 Mineral Composition and Nutritional Relevance

Mineral content in rose petals and roselle calyces varies considerably with cultivar, soil, irrigation, and tissue type; Table S4 (Kazaz et al. 2009; Mlcek et al. 2021; Salami and Afolayan 2021) shows a variability that matters here specifically because mineral fortification claims depend on which plant part and cultivar underlie the reported value.

Potassium is consistently dominant in both species, supporting osmotic regulation and cardiovascular function (Stone et al. 2016); calcium and magnesium are present at physiologically meaningful concentrations, with roselle calcium reaching 2105 mg/100 g DM in some cultivars (Riaz et al. 2020) a concentration that can contribute meaningfully to calcium intake, though this concentration reflects dried plant material instead of a realistic single serving; translating it into an actual dietary contribution toward the 1000–1200 mg/day adult requirement (National Institutes of Health Office of Dietary Supplements 2025) would require establishing typical consumption volume and bioavailability, neither of which is currently established. Roselle's iron content is where composition data alone becomes actively misleading without a bioavailability caveat attached.

Reported iron concentrations look nutritionally promising on paper, but plant‐derived iron is predominantly non‐heme (Fe^3^^+^), and its absorption depends heavily on co‐occurring compounds. The ascorbic acid naturally present in roselle may promote non‐heme iron absorption by facilitating the reduction of ferric to ferrous iron. However, this potential benefit may be substantially limited in roselle‐based beverages, where polyphenol–iron interactions have been reported to reduce iron bioavailability, with only approximately 25% of added iron remaining bioaccessible (Oyewole and Diosady 2023). This means a roselle iron‐fortification claim based on raw mineral content, without addressing this complexation, would substantially overstate delivered nutritional value; the composition figure and the functional outcome are not the same number, and treating them as interchangeable is the practical error. For product development, this has an important practical implication; listing how much mineral is in the ingredient (Table S4) does not mean the body will actually absorb that amount. To claim that a product ‘fortifies’ with a mineral, it has to show that the mineral stays available for absorption in a specific food, at the serving size, and after all processing steps (mixing, baking, storage, etc.). The studies summarized in the composition literature report mineral amounts, but they do not, by themselves, prove that the mineral is bio accessible in the final product.

### 2.6 Organic Acids and Their Functional Implications

Organic acids shape sourness, pH, microbial stability, anthocyanin preservation, and mineral chelation in both plants (Table S5), but differ enough in acid profile and concentration that their functional and safety implications diverge in ways worth stating explicitly.

Rose petals contain fewer organic acids than roselle. Malic and citric acid each ≈1.2 g/100 g DM, quinic acid highest at 1.5 g/100 g DM, oxalic acid at 0.3 g/100 g DM (Pires et al. 2017). Values vary substantially by cultivar; however, malic acid alone spans 0.23–6.08 mg/g FM across garden *Rosa* cultivars, a 26‐fold range (Vukosavljev et al. 2023), meaning no single typical profile applies without specifying cultivar. Consequently, acid‐dependent formulation decisions, such as pH modulation and anthocyanin stabilization via chelation, cannot be transferred between rose cultivars without verification. Echoing the standardization concerns raised elsewhere in this review.

Rose's ascorbic acid content, while modest in absolute terms, has been reported to exceed that of blueberry, raspberry, red currant, and strawberry (Vukosavljev et al. 2023). This notable finding, although based on a single comparative study instead of a systematic survey, should be considered promising but not yet established until independently replicated. Oxalic acid at 0.3 g/100 g DM (Pires et al. 2017) is low relative to roselle but not negligible, since it chelates calcium regardless of concentration, raising the same dairy‐compatibility and mineral‐bioavailability concerns already noted for roselle (Section 3.1). This is a shared constraint across both plants, not a roselle‐specific Roselle's organic acid profile is dominated by malic and tartaric acid (Izquierdo‐Vega et al. 2020), at concentrations high enough that formulation constraints become the more relevant framing than nutritional benefit alone. The same acids that give roselle its sourness and support anthocyanin and color stability also chelate calcium and reduce dairy protein solubility, and oxalate specifically carries a documented kidney‐stone risk in susceptible individuals (Al‐Wandawi 2015; Bargagli et al. 2020).

This safety concern is addressed in Section 3.1 but should be read alongside the functional acidulant value described here. Roselle's organic acids offer both functional value and a formulation constraint at once, arising from the very same compounds. This dual character is the central practical takeaway for its acid profile, more informative than any single reported concentration taken in isolation. The shared mechanism of sustained low pH exposure in both rose‐ and roselle‐based products means that formulation guidance for mitigating dental erosion risks applies equally to both botanicals (Hughes et al. 2000).

### 2.7 Comparative Functional Perspective

A comparative evaluation of rose and roselle reveals that these two plants fulfill fundamentally different but complementary technological roles within food systems. From a biochemical perspective, rose petals are characterized by elevated concentrations of flavonol glycosides, anthocyanins, volatile terpenoids, phenolic acids, and aromatic alcohols, which primarily contribute to floral aroma complexity, sensorial quality, and oxidative protection. In contrast, roselle calyces are dominated by anthocyanins, organic acids, and hydroxycinnamic acid derivatives that confer intense red pigmentation and pronounced acidity. Consequently, rose is primarily utilized for aromatic, sensorial, and cosmetic applications, whereas roselle functions predominantly as a natural colorant and acidifying ingredient.

This distinction has important implications for industrial product development. Rose‐derived ingredients are particularly suited to premium sensory applications including beverages, confectionery, fermented dairy systems, and cosmetics where aroma complexity and floral identity are central quality attributes. Roselle exhibits broader techno‐functional applicability through its simultaneous capacity to provide natural coloration, acidity, viscosity modification, and nutritional enhancement.

## 3 Food Safety, Allergenicity, and Industrial Standardization Considerations

### 3.1 Natural Toxic Compounds

In rose, safety conclusions cannot be generalized across species, cultivars, and product forms. Cytotoxic activity reported in cell‐based assays reflects experimental conditions and does not necessarily indicate a hazard from consuming whole petals (Hegde et al. 2022; Nowak et al. 2014), while no characteristic endogenous toxin has been identified. Saponins and anthraquinones have been detected in rose tissues, but their concentration and toxicological relevance remain uncharacterized (Chukwu et al. 2019; Mallick et al. 2024), a gap worth resolving given that hydroxyanthracene derivatives in other plant genera carry documented laxative and genotoxicity concerns (EFSA Panel on Food Additives and Nutrient Sources added to Food (ANS) et al. 2018). These constituents are best treated as bioactive or potentially antinutritional pending further characterization, not established toxicants. In roselle, cyanogenic compounds are the most plausible direct concern. Calyx hydrocyanic acid levels are low (0.16 mg/100 g red, 0.13 mg/100 g green DM) and cultivar‐dependent (Adanlawo and Ajibade 2006), EFSA has established an acute reference dose of 20 µg/kg body weight for dietary cyanide from any source (EFSA Panel on Contaminants in the Food Chain (CONTAM) et al. 2019), though translating the calyx concentration into an actual dose relative to this reference would require establishing typical serving size and consumption pattern, which are not currently available.

However, existing data derive largely from leaf instead of calyx tissue, cyanide content varies with maturity and processing (Musa and Ogbadoyi 2012), and the literature does not consistently distinguish free cyanide from total cyanogenic potential. Roselle's cyanogenic risk is therefore tissue‐ and dose‐dependent instead of being a fixed species trait, and no validated intake limits exist (Ali et al. 2005; Joint FAO/WHO Expert Committee on Food Additives 2012).

Saponins, phytates, tannins, and oxalates should not be treated as toxicants by default; saponin evidence remains compositional only (Chukwu et al. 2019; Mallick et al. 2024), phytate and tannin are more accurately antinutrients affecting mineral bioavailability (Salami and Afolayan 2021), and oxalate‐related risks are addressed in Section 2.6. Cyanogenic glycosides and anthraquinones warrant toxicological evaluation; phytate and tannin are better managed through processing such as fermentation or roasting (Samtiya et al. 2020).

Roselle is generally well tolerated at studied exposures, though acute toxicity data for calyx extract (LD_50 _> 5000 mg/kg (Ali et al. 2005) should not substitute for repeated‐exposure evidence, given documented hepatic, renal, and reproductive effects under high‐dose preclinical exposure and herb–drug interactions, including chloroquine pharmacokinetics (Muhamad Rosli et al. 2026). Alkaloid contamination via field cross‐contact or pollination, documented in chamomile (Fernández‐Pintor et al. 2025), remains uncharacterized for rose and roselle specifically, an unresolved evidence gap.

### 3.2 Microbial Safety

Microbial safety is a concern for edible flowers and may arise from pre‐harvest sources and post‐harvest handling, particularly relevant to freshly consumed rose petals and roselle, creating opportunities for contamination and cross‐contamination (Matyjaszczyk and Śmiechowska 2019). Contamination has been documented in rose‐derived products, including *R. damascena* rose water, where bacterial contamination was associated with processing and storage conditions (Abidi et al. 2021). This limited microbiological evidence should not be interpreted as evidence of low risk; instead, it indicates a need for direct microbiological characterization of fresh edible rose petals.

Traditional roselle beverages such as Zobo, Bissap, and Karkade are prepared by boiling dried calyces, providing an inherent thermal microbial reduction step. Cold extraction and low‐temperature processing used to preserve heat‐sensitive anthocyanins (Section 4.1) may reduce microbial inactivation, creating a trade‐off between phytochemical retention and microbial safety. Similarly, reuse of blanching or extraction water (Section 4.4) for compound recovery should be evaluated for potential microbial accumulation before industrial application. These concerns are not unique to rose or roselle but are relevant when developing minimally processed, anthocyanin‐rich products.

Dried roselle calyces and Zobo juice have been reported to contain bacterial and fungal contaminants, including *Staphylococcus aureus, Escherichia coli, Bacillus* spp., *Aspergillus*, and *Penicillium* (Omemu et al. 2006). Similar contamination has been observed in street‐ vended *H. sabdariffa* has shown mesophilic aerobic microbial counts of 1.4–1.5 × 10^5^ CFU/mL, though *E. coli*, total coliforms, and *Salmonella* were not detected in the same samples (Ahon et al. 2022; Carole et al. 2022), highlighting the importance of hygienic processing and storage even where specific pathogens are absent. More systematic surveillance is needed for fresh rose petals and roselle materials, particularly as production shifts from traditional to larger‐scale processing.

### 3.3 Pesticide Residues and Chemical Contaminants

Pesticide residues are a relevant safety concern for edible rose, partly because rose is produced for both ornamental and food use. Ornamental production may involve pesticides not intended for edible crops. Accordingly, a 2022 RASFF notification reported lufenuron, chlorpyrifos, and imidacloprid in rose blossoms from Pakistan (RASFF Window 2022), while ornamental roses from Belgium contained an average of 14 pesticide residues per sample (Toumi et al. 2016). Although these findings cannot be directly translated into dietary risk, they emphasize the need for dedicated food‐grade supply chains and traceability.

Direct evidence from edible roses confirms the need for residue monitoring. In 54 edible rose samples, 74 pesticides were detected, and 91% of identified compounds were not registered for edible roses. Banned or restricted substances were also found (Bian et al. 2026). Drying may further increase exposure by concentrating pesticide residues approximately 3.0 to 3.9‐fold, although dietary risk is compound‐specific (Qin et al. 2025). Thus, processing can improve product stability while simultaneously increasing pesticide exposure when residues are present.

Comparable pesticide‐residue data for roselle remain scarce, representing an evidence gap. Validated multi‐residue methods, such as QuEChERS coupled with HPLC‐MS/MS, demonstrate that systematic surveillance is feasible (Bian et al. 2026). Roselle intended for human consumption should therefore receive similar attention. EU legislation includes roselle and rose within MRL frameworks, while Regulation (EC) No 396/2005 requires processing‐related changes in residue levels to be considered. However, species‐ and process‐specific residue data, particularly for dried products, remain limited.

### 3.4 Allergenicity

Allergenicity remains poorly characterized for rose and roselle. Rose has been associated with Immunoglobulin E (IgE) mediated occupational and environmental sensitization. For example, 19% of rose cultivators in Turkey showed atopy and specific IgE reactivity to *Rosa rugosa* (Demir et al. 2002), and one case of anaphylaxis following exposure to rose blossom dew has been reported (Karakaya and Kalyoncu 2003).

However, these studies primarily reflect inhalational or occupational exposure instead of consumption. The available evidence is also limited and dated, with little information on oral exposure, dose, or food matrix. Current data therefore demonstrate allergenic potential under specific exposure conditions but do not establish a comparable risk from consuming rose‐containing foods.

For *H. sabdariffa*, allergenicity data are even more limited. No dedicated occupational, dietary, or clinical studies were identified, and consumer warnings about possible allergic reactions appear to be precautionary, not supported by species‐specific clinical evidence (WebMD 2026). Consequently, the allergenic potential of roselle remains largely unresolved. This evidence gap is particularly relevant for roselle seed protein, which has been proposed as a functional ingredient because of its high protein content, reaching up to 33.45% DM, and favorable emulsifying and water‐binding properties (Mawouma et al. 2024; Tounkara et al. 2014). As seed storage proteins including several major classes of food allergens (Breiteneder and Radauer 2004). The absence of allergenicity characterization warrants attention before broader use of concentrated roselle protein ingredients. Importantly, limited reports of allergic reactions should not be interpreted as evidence that roselle is allergen‐free.

Overall, available evidence for rose and roselle products does not indicate a widespread allergenicity concern associated with traditional consumption. However, species‐specific data remain limited, particularly for concentrated extracts or protein‐rich ingredients intended for new food applications. Further characterization would therefore support more robust risk assessment and communication as these ingredients move beyond traditional uses.

### 3.5 Legislation and Regulatory Framework

Regulatory treatment of rose and roselle remains incomplete and poorly aligned with their current food uses. No authoritative international list of edible flowers or recommended intake levels has been established (Fernandes et al. 2017; Nicolau 2016). WHO Codex Alimentarius provides a framework for harmonizing standards for dried spices and culinary herbs, including floral products such as cloves and saffron, neither rose nor roselle is currently covered by the Committee on Spices and Culinary Herbs (CCSCH) (Codex Alimentarius Commission 2026a, 2026b). This suggests a gap in regulatory prioritization, not institutional capacity, and leaves safety assessment and market authorization largely dependent on individual jurisdictions.

The EU regulates specific aspects of rose and roselle use, not the plants comprehensively. Both *H. sabdariffa* and *Rosa* spp. are recognized food commodities under pesticide MRL legislation, yet no EU‐wide rules specifically govern their production, harvesting, or storage (Matyjaszczyk and Śmiechowska 2019; Regulation (EU) 2018/62 2018; Regulation (EU) 2022/476 2022). While traditional forms of rose and roselle foods are not novel foods, substantially modified extracts or protein isolates (Section 2.2) may require assessment under European Parliament and Council of the European Union, 2015. Importantly, EFSA's consideration focuses on specific plant part, form, and production process, not on parent species' consumption history (EFSA Panel on Nutrition, Novel Foods, and Food Allergens (NDA) et al. 2024).

The US regulates specific extractive forms, not whole species. Rose oil, absolute, and rose water have Generally Recognize as Safe (GRAS) status as flavorings (FEMA Nos. 2988–2990), as *H. sabdariffa* blossom extract (Cohen et al. 2020). China similarly recognizes rose oil, absolute, and concrete as flavoring substances, but lists no equivalent roselle ingredient (National Health Commission of the People's Republic of China 2024). Across these systems, rose receives more consistent species‐specific recognition, while the regulatory status of functional ingredients such as seed protein isolates remains less clearly defined.

No comprehensive regulatory framework was identified for major traditional consumption regions in West Africa and South/Southeast Asia, despite widespread roselle products such as Zobo, Bissap, and Karkade (Section 4.1). Regulatory attention appears to prioritize export‐oriented and high‐value processed products over traditional domestic uses (Section 2.2).

Overall, regulation remains fragmented by product format and jurisdiction, particularly beyond conventional teas, flowers, and flavorings. Greater harmonization could be achieved by explicitly incorporating edible flowers such as rose and roselle into existing international standard‐setting mechanisms, including the CCSCH.

### 3.6 Post‐Harvest Handling, Traceability, and Industrial Standardization

Traditional post‐harvest practice remains poorly standardized and inconsistently implemented. FAO guidance recommends preventing roselle calyces from contacting the ground; they are commonly dried on plastic sheets placed directly on the ground, increasing insect infestation and mold (FAO 2004). As noted in the microbial safety (Section 3.2), this reflects a gap in compliance and governance, not in knowledge.

Where quality specifications exist, they are largely buyer‐specific. FAO documentation shows that commercial importers define their own limits for moisture, ash, and microbial parameters, not following an industry‐wide standard (FAO 2004). Thailand stands out for its organized production and reputation for cleanliness, which has supported greater value‐added processing.

Formal quality standards exist for high‐value rose products. ISO 9842 defines quality criteria for *R. damascena* essential oil from Turkey, Morocco, and Bulgaria, including a GC‐based authenticity assessment (Mykhailenko 2024). However, the standard covers only a limited set of reference compounds and does not include newer authenticity markers such as enantiomeric terpenes (Raeber et al. 2023). Its scope is also restricted to essential oil from three origins. No comparable standard exists for rose petals used as food ingredients or for roselle products, highlighting substantial gaps beyond established perfumery applications.

Authentication challenges differ between genus, species, and cultivar. Roselle products show substantial uncontrolled compositional variation (Li et al. 2024), despite their characteristic anthocyanin profile being sufficiently distinctive to detect fraud in other botanicals (Gafner et al. 2023). In contrast, rose essential oil itself is vulnerable to economically motivated adulteration, including dilution with cheaper oils or undeclared terpene addition (Gafner et al. 2023). Thus, roselle's primary priority requires improved compositional standardization and enforcement, whereas rose requires stronger adulteration screening.

Shelf‐life data remain compound‐ and product‐specific. Roselle anthocyanins are heat‐ and storage‐sensitive (Section 4.1), while drying concentrate pesticide residues in rose (Section 3.3), yet these effects are not incorporated into harmonized shelf‐life or grading criteria. Moisture limits remain buyer‐specific despite their importance for microbial and chemical stability.

Overall, the evidence points less to a lack of technical knowledge than to a failure to translate existing specifications, markers, and stability data into harmonized and enforceable quality standards.

## 4 Product Development and Industrial Innovations

### 4.1 From Traditional Beverages to Standardized Functional Systems

The global functional beverage sector's projected 7.93% growth at a compound annual growth rate (CAGR) through 2031 (Mordor Intelligence 2026), has created commercial space for rose‐ and roselle‐based products, but market expansion alone does not resolve the technical barriers identified in this review; it heightens the imperative to address them. Traditional preparations (Zobo, Bissap, Karkade) are shifting toward industrially standardized formulations, and rose tea, liqueurs, and wine additives are each supported by physicochemical data (Ścibisz et al. 2017; Seong et al. 2017). This confirms technical feasibility, not industrial scalability, which is constrained primarily by cultivar‐dependent phytochemical instability, not generic processing challenges. In rose‐based beverages, cultivar identity and petal water status govern anthocyanin, carotenoid, and flavonoid retention: dry petals and cv. *Rubra* outperform fresh petals and cv. *Alba* in *R*. rugosa liqueur (Fascella et al. 2022). Raw‐material specification is thus a critical formulation variable (Section 2). White rose petal extract added to wine at 0.10%–0.25% (*w/v*) increased polyphenols and antioxidant activity dose‐dependently without impairing fermentation or sensory acceptability (Seong et al. 2017), indicating that rose can function as a bioactive enrichment ingredient, although evidence is currently limited to wine and cannot be generalized across beverage types. Dried roselle powder reaches anthocyanin concentrations up to 310.5 mg/100 g DM (Gartaula and Karki 2013), but high‐temperature pasteurization degrades this value by 25%–56% within 60 min, far faster than comparable losses reported in Section 2 (Suzery et al. 2020). Thermal protocols validated on other matrices therefore cannot be assumed transferable to roselle; industry must instead adopt milder alternatives such as cold extraction, low‐temperature or pulsed‐electric‐field pasteurization, and membrane filtration.

This shift, however, is not a costless improvement: replacing traditional boiling with cold processing eliminates the inherent thermal microbial safeguard of conventional Zobo, Bissap, and Karkade preparation (Section 3.2), representing a safety process trade‐off that the current literature has not yet resolved. Similarly, the extraction‐yield‐versus‐thermal‐stability tension already identified in Section 2.4.1 applies directly here; conditions that maximize anthocyanin recovery at extraction do not necessarily preserve that anthocyanin through to the finished beverage. The future competitiveness of rose‐ and roselle‐based beverages therefore depends on stabilization technologies that address extraction yield, thermal stability, and microbiological safety simultaneously a three‐way constraint this literature has so far only addressed pairwise.

### 4.2 Bakery and Confectionery Systems: Thermal Stress and Matrix Engineering

Incorporating rose and roselle powders into bakery products constitutes an evidence‐based strategy for phytochemical enrichment, supported by compositional and functional data, not hypothetical benefit. Fortification with 5% rose petals enhanced polyphenols, flavonoids, DPPH radical‐scavenging activity, and anthocyanins in biscuits without compromising acceptability (Sik et al. 2024), while roselle calyx fortified muffins attained quantifiable anthocyanin (126.63 mg Cyn‐3‐glu/100 g), phenolic (12.91 mg GAE/100 g), and ascorbic acid (12.10 mg/100 g) levels alongside sensory scores up to 84% (Marak et al. 2022). These improvements are realized under baking conditions (150–170°C) that concurrently promote anthocyanin degradation, phenolic oxidation, and Maillard reactions (Fallico et al. 2022; Panghal et al. 2018), framing fortification as a kinetic contest between bioactive enrichment and thermal degradation, not mere additive incorporation.

*Rosa* spp. powders show greater thermal compatibility because their functional value depends on relatively heat‐stable flavonols and aroma compounds, not anthocyanins (Gül and Tekeli̇ 2019; Hnin et al. 2021). This follows from the compositional divergence outlined in Section 2.7, not from a coincidental processing observation. Roselle has no equivalent buffer, such as in fortified muffins; antioxidant activity decreased from 27.30% in batter to 12.53% after baking (≈54% loss), indicating that standard baking conditions are fundamentally incompatible with anthocyanin and phenolic preservation as currently applied (Marak et al. 2022). Drying and powdering roselle calyces improve shelf life and bioaccessibility, but the required drying temperatures degrade anthocyanins and phenolics before baking begins (M'be et al. 2023). Roselle's fiber fraction adds a second, independent constraint, such as an increase in dough water absorption and toughness while reducing extensibility, generally increasing hardness (Gómez et al. 2003). This structural effect is separate from phytochemical degradation, so roselle incorporation requires optimizing two largely independent variables like bioactive retention and dough rheology simultaneously.

Encapsulation partially addresses roselle anthocyanin instability. Spray‐dried roselle anthocyanins with maltodextrin plus gum Arabic walls achieved encapsulation efficiencies up to 99.87%, and composite walls (β‐cyclodextrin plus proteins) further improved thermal stability versus single‐wall systems (Li et al. 2022; Nguyen et al. 2022). Protein‐polysaccharide or lipid‐based encapsulation also reduces evaporative loss of rose's volatile aromatics (Saifullah et al. 2019). These are quantified gains in bioactive retention, but they do not address the separate dough‐rheology constraint from roselle fiber; encapsulation alone therefore does not resolve roselle's full bakery formulation challenge.

### 4.3 Colloidal Stabilization and the Pectin‐Anthocyanin Matrix

Roselle's value in gel‐based systems rests on synergy between its endogenous pectins and anthocyanins, which interact functionally rather than simply co‐exist. Pectin networks may reduce anthocyanin mobility, limit oxidative exposure, and maintain the acidic microenvironment favoring the stable red flavylium cation form (Zang et al. 2022), consistent with the pH‐dependent stability mechanism established in Section 2.4.3 and suggesting active protection by roselle's matrix. Whether this translates into demonstrated shelf‐life extension beyond the conventional 6–12‐months window for fruit‐based jams, or remains a plausible mechanistic inference, requires confirmation (Ochelle et al. 2024). Beyond pigment stabilization, roselle pectins enhance gel elasticity, water‐binding, and mouthfeel, enabling one ingredient to deliver color, thickening, acidification, and antioxidant functions for clean‐label formulations.

Rose contributes to the same product category as roselle, but in a fundamentally different way, not just to a lesser extent. Products such as Gulkand, rose jams, and rose‐infused gelato show that rose anthocyanins and phenolics can enhance sensory acceptance and antioxidant activity without negatively affecting texture (Kanse et al. 2020). However, this is primarily a sensory and antioxidant contribution, not a structural one.

Because rose has a comparatively low endogenous pectin content, it cannot reproduce roselle's simultaneous thickening and stabilizing functions. This functional asymmetry, first described at the compositional level in Section 2.7 (rose as an aromatic/sensory contributor, roselle as a structural/functional contributor), is now confirmed at the level of finished gel‐based products, not only in raw material composition.

What remains unresolved is mechanistic, not applied. The molecular interactions among anthocyanins, pectins, sugars, and proteins in these matrices have not yet been characterized in sufficient detail to enable rational, instead of empirical (trial‐and‐error), formulation design. This represents a distinct knowledge gap relative to the functional outcomes demonstrated above.

### 4.4 Botanical Acidulants and Processing Applications

Rose provides mild background acidity, whereas roselle's stronger organic acid profile drives sourness, flavor enhancement and microbial stabilization (Al‐Wandawi 2015; Pires et al. 2017). This is the same asymmetry this review has identified repeatedly across composition, extraction, and now application; *Rosa* spp. contributes sensory nuance; roselle contributes functional substance.

Rose cultivar pigment variability (yellow: low anthocyanin/high carotenoid; pink: high flavonol; orange: high anthocyanin/low flavonol) directly guides application (Wan et al. 2019). Red/pink cultivars suit pigmented, antioxidant‐forward products; pale/yellow cultivars suit flavor‐forward, minimally pigmented applications.

Processing introduces a shared constraint across both plants, not a species‐specific one. For *Rosa* spp., hot‐air drying above 70°C degrades phenolic content and antioxidant activity, while freeze‐drying better preserves both compounds and color (Baibuch et al. 2023); for roselle, the equivalent constraint applies through blanching and thermal softening. In both plants, the same underlying trade‐off recurs; blanching, necessary to inactivate enzymes and improve processability, simultaneously leaches water‐soluble phenolics, organic acids, and minerals into processing water (Severini et al. 2016), a single phenomenon affecting both plants identically, not two separate findings.

Recovering this stream for reuse in beverages offers sustainability benefits for rose and roselle, but its extraction efficiency, pesticide residues, and microbial safety remain insufficiently evaluated.

Roselle's green and red calyces diverge functionally in a way that mirrors, but does not duplicate, the rose cultivar distinction above: green calyces, lower in anthocyanin and milder in flavor, suit savory applications where excessive pigmentation is undesirable, while red calyces suit beverages and sauces requiring intense color and pronounced acidity (Li et al. 2022; Salinas‐Moreno et al. 2024). The parallel structure across both plant material for flavor use and pigmented material for color use suggests this is a general compositional principle for these two botanicals.

### 4.5 Cosmetic and Dermaceutical Applications: From Luxury Ingredients to Functional Bioactives

Cosmetic use of rose and roselle is shifting from purely sensorial, luxury‐oriented positioning toward evidence‐based dermaceutical claims, though the strength of that evidence varies considerably by claim. This section treats that variation explicitly instead of presenting all findings as equally established.

Rose water and petal extracts are rich in flavonoids, tannins, triterpenoids, and volatile terpenes with plausible antioxidant and anti‐inflammatory relevance to skin protection (Safia et al. 2019), consistent with the flavonol‐dominant phytochemical profile already established for rose in Section 2.4. Efficacy is dose‐dependent, and a single formulation study identified 63‐65°C as compatible with preserving color, aroma, and stability in a low‐pH lip balm system (Kore et al. 2022), a useful data point, but a narrow one; it establishes feasibility for one formulation type at one temperature range, not a general processing window for rose in cosmetics. Standardization remains the binding constraint here, for the same reason established throughout this review: phenolic and volatile composition varies by cultivar, extraction method, and storage condition (Sections 2.4 and 3.5), so reproducible cosmetic efficacy requires chemically standardized extracts rather than raw botanical material specified by species name alone.

Roselle seed oil represents a distinct opportunity from rose, with its favorable unsaturated fatty acid and tocopherol profile supporting oxidative and thermal stability. Extraction below 110°C for 25 min yields approximately 22% oil, while the formulated oil demonstrates high thermal stability already established in Section 2.3. Roselle seed oil may offer advantages over conventional seed oils in hot‐process cosmetics, functioning as a carrier for lipophilic actives (Akinoso and Suleiman 2011; Eisa et al. 2024; Kitprathaung et al. 2023). However, evidence remains limited. Roselle's anthocyanins offer a clean‐label alternative to synthetic red dyes in lip products and tinted moisturizers, contributing both color and measurable antioxidant activity against UV‐induced free radicals, with reported stability under moderate cosmetic‐manufacturing and storage temperatures (Ariestanti et al. 2023). Roselle anthocyanin stability depends on pH, temperature, oxygen, and light; therefore, food‐validated stabilization strategies remain relevant for cosmetic formulations.

## 5 Conclusion and Future Perspectives

The integration of rose and roselle into food, nutraceutical, and cosmetic applications represents a significant opportunity in the growing market for possible clean‐label, plant‐derived ingredients. Both species provide multifunctional value through phenolics, anthocyanins, volatile compounds, polysaccharides, proteins, lipids, minerals, and organic acids. Rose is particularly valuable for aromatic volatiles and flavonol‐rich antioxidant activity in beverages, confectionery, and cosmetics, whereas roselle offers broader techno‐functional versatility through anthocyanins, acidity, pectins, minerals, and seed lipids, functioning as a colorant, acidulant, stabilizer, and nutraceutical ingredient.

Industrial standardization is constrained less by compositional knowledge than by unresolved safety, allergenicity, extraction, regulatory, and post‐harvest gaps (Section 3). Anthocyanin instability during processing and storage further limits product consistency (Section 4). The absence of harmonized quality standards for dried petals, calyces, and derived products restricts reliable assessment of contamination, authenticity, and shelf stability. Thus, broader industrial adoption requires standardized safety characterization, validated methodologies, and regulatory clarity.

Future research should prioritize plant‐part‐ and dose‐specific toxicological and allergenicity assessments, particularly for roselle calyces and seed proteins, alongside microbiological surveillance (Section 3). Extraction protocols require cross‐laboratory validation to balance yield with phytochemical preservation, while regulatory harmonization should clarify edible‐flower standards and the novel‐food status of extracts and protein isolates (Section 3.5). Post‐harvest standardization, traceability, and authentication also require integration into food‐relevant industry standards (Section 3.6). Encapsulation, non‐thermal preservation, controlled fermentation, and matrix engineering remain important for improving phytochemical retention and scalability (Section 4). However, their industrial value depends on resolving the underlying safety and standardization gaps. Future development should also emphasize cultivar selection and sustainable valorization of underutilized fractions, particularly roselle seeds and processing by‐products, while accounting for compositional variability (Section 2).

## Author Contributions

**Temilola Dada**: conceptualization, writing – original draft, writing – review and editing. **Susanne Neugart**: writing – review and editing, supervision. **Layla Engelhardt**: supervision, writing – review and editing.

## Conflicts of Interest

The authors declare no conflicts of interest.

## Supporting information




**Supplementary Material**: jfds71524‐sup‐0001‐TableS1‐S6.docx

## Data Availability

No data were used for the research described in the article.
