# Supplementary material for: Edible Rose and Roselle: A Critical Review of Nutritional Composition, Safety, and Regulatory Considerations for Product Development
Source: J Food Sci. 2026 Oct 19;91(10):e71524. doi: 10.1111/1750-3841.71524 (PMC13678837; doi:10.1111/1750-3841.71524)
Supplement: Supplementary file 1 [file JFDS-91-0-s001.docx]

**Table S1.** The table summarizes the energy, macronutrients (carbohydrates, protein, fat, fiber, and ash) and sugars (glucose, fructose, and sucrose) reported for edible *Rosa* spp.

| **Plant/part** | **Nutritional components** | **Specie** | **Amount** | **Reference** |
| --- | --- | --- | --- | --- |
| Rose petal | Carbohydrates | *Rosa* spp. | 60-86% DM | (Hegde et al., 2022) |
| Rose petal | Carbohydrates | *Rosa* spp. | 73.3 - 75.8% DM | (Liu et al., 2024) |
| Rose petal | Crude Protein | *Rosa* spp. | 2.5 - 12.2 % DM | (Hegde et al., 2022) |
| Rose petal | Protein | *Rosa* spp. | 4.3 g DM | (Pires et al., 2017) |
| Rose petal | Protein | *Rosa* spp. | 12.6 - 14.2% DM | (Liu et al., 2024) |
| Rose petal | Crude Protein | *Rosa* spp. | 2.9 g/kg FM | (Mlcek et al., 2021) |
| Rose petal | Crude Protein | *Rosa* spp. | 7.6 g/100g DM | (Pires et al., 2017) |
| Rose petal | Crude Fat | *Micrantha, canina, and damascena* | 1.3 - 3.3 g DM | (Hegde et al., 2022) |
| Rose petal | Total Fat | *Rosa* spp. | 4 % DM | (Hegde et al., 2022) |
| Rose petal | Total Fat | *Rosa damascena* | 2.0 g/100g DM | (Pires et al., 2017) |
| Rose petal | Fat | *Rosa* spp. | 3 - 5% DM | (Liu et al., 2024) |
| Rose petal | Energy | *Grandiflora, canina, and micrantha* | 388 - 465 kcal/100g DM | (Hegde et al., 2022) |
| Rose petal | Ash | *Rosa* spp. | 4.3 g DM | (Pires et al., 2017) |
| Rose petal | Fiber | *Rosa grandiflora* | 3.20% | (de Lima Franzen et al., 2019) |
| Dried rose extract | Glucose | *Rosa damascena* | 85 mg/g Extract | (Trendafilova et al., 2023) |
| Dried rose extract | Fructose | *Rosa damascena* | 108 mg/g Extract | (Trendafilova et al., 2023) |
| Dried rose petals | Fructose | *Rosa* spp. | 5.1 g/100g DM | (Pires et al., 2017) |
| Dried rose petals | Glucose | *Rosa* spp. | 3.2 g/100g DM | (Pires et al., 2017) |
| Dried rose petals | Sucrose | *Rosa* spp. | 2.4 g/100g DM | (Pires et al., 2017) |

DM: Dry matter

**Table S2.** The table summarizes the energy, macronutrients (carbohydrates, protein, fat, fiber, and ash) and sugars (glucose, fructose, and sucrose) reported for different part of roselle

| **Plant/part** | **Nutritional components** | **Specie** | **Amount** | **Reference** |
| --- | --- | --- | --- | --- |
| Roselle calyx | Carbohydrates | *Hibiscus sabdariffa* L. | 60% DM | (Kholaf, 2024) |
| Roselle seeds | Carbohydrates | *Hibiscus sabdariffa* L. | 21.7% | (El-Deab & Ghamry, 2017) |
| Roselle calyx | Crude Protein | *Hibiscus Rosa sinensis* L | 3.9 g DM | (Hegde et al., 2022) |
| Roselle calyx | Crude Protein | *Hibiscus sabdariffa* L. | 5.7 - 5.8% DM | (Kholaf, 2024; Salami & Afolayan, 2021) |
| Red roselle calyx | Crude Fat | *Hibiscus sabdariffa* L. | 0.2% - 1.2% DM | (Kholaf, 2024; Salami & Afolayan, 2021) |
| Roselle pre-flowering green | Crude Fat | *Hibiscus sabdariffa* L. | 3.72% DM | (Salami & Afolayan, 2021) |
| Roselle pre-flowering red | Crude Fat | *Hibiscus sabdariffa* L. | 2.9% DM | (Salami & Afolayan, 2021) |
| Calyx green | Crude Fat | *Hibiscus sabdariffa* L. | 1.7% DM | (Salami & Afolayan, 2021) |
| Green roselle seed | Crude fat | *Hibiscus sabdariffa* L. | 13.1% DM | (Salami & Afolayan, 2021) |
| Red roselle seeds | Crude Fat | *Hibiscus sabdariffa* L. | 11.5% DM | (Salami & Afolayan, 2021) |
| Roselle calyx | Crude Fiber | *Hibiscus Rosa sinensis* L | 15.8% / 13.6% DM | (Izquierdo-Vega et al., 2020; Kholaf, 2024; Salami & Afolayan, 2021) |
| Roselle calyx | Crude Fiber | *Hibiscus Rosa sinensis* L | 13.6% - 15.8% DM | (Kholaf, 2024; Salami & Afolayan, 2021) |
| Roselle seeds | Crude Fiber | *Hibiscus sabdariffa* L. | 16.25% | (El-Deab & Ghamry, 2017) |
| Roselle calyx | Energy | *Hibiscus sabdariffa* L. | 912 KJ DM | (Salami & Afolayan, 2021) |
| Roselle seed | Energy | *Hibiscus sabdariffa* L*.* | 1018 KJ DM | (Salami & Afolayan, 2021) |
| Roselle calyx | Ash | *Hibiscus sabdariffa* L. | 10.9 % - 11.3 % DM | (Kholaf, 2024; Salami & Afolayan, 2021) |
| Roselle seed | Crude Protein | *Hibiscus sabdariffa* L. | 22 - 22.5 % DM | (Ghislain et al., 2014; Mahmoud et al., 2024; Salami & Afolayan, 2021) |
| Roselle seed | Crude Protein | *Hibiscus sabdariffa* L. | 33.45 % DM | (Hainida et al., 2008) |
| Roselle seed | Crude Fiber | *Hibiscus sabdariffa* L. | 20 % DM | (Ghislain et al., 2014; Mahmoud et al., 2024) |
| Roselle seed | Lipid | *Hibiscus sabdariffa* L. | 21 % DM | (Ghislain et al., 2014; Mahmoud et al., 2024) |
| Roselle seed | Ash | *Hibiscus sabdariffa* L. | 5.7-6.4 % DM | (El-Deab & Ghamry, 2017; Ghislain et al., 2014; Mahmoud et al., 2024) |
| Roselle calyx | Glucose | *Hibiscus sabdariffa* L. | 1.1 % DM | (Riaz et al., 2020) |
| Roselle calyx | Fructose | *Hibiscus sabdariffa* L. | 0.3 % DM | (Riaz et al., 2020) |
| Roselle calyx | Sucrose | *Hibiscus sabdariffa* L. | 0.5 % DM | (Riaz et al., 2020) |

DM: Dry matter

**Table S3.** Some predominant phytochemical composition of rose and roselle.

| Plant | Content | Compounds | Concentrations | Reference |
| --- | --- | --- | --- | --- |
| *Rosa hybrida* | Anthocyanin | Cyanidin 3,5-*O*-diglucoside, Pelargonidin 3,5-*O*-diglucoside | 375mg/100 g DM,  31.2 mg/ 100 g DM | (Kumari et al., 2021; Lee et al., 2011) |
| *Rosa damascena* petals | Phenolics | Quercetin  Chlorogenic acid  Rutin | 7.3 µg/g DM  126 µg/g DM  7.4 µg/g DM | (Shameh et al., 2018) |
| *Rosa rugosa* petals | Phenolics | Myricetin 3,5-di-*O*-glucoside,  Quercetin 3,4-di-*O*-glucoside,  Kaempferol 3,4-di-*O*-glucoside,  Quercetin 3-*O*-glucosyl-xyloside, and  Ellagic acid | 79.0 mg/100 g FM  164.6 mg/100 g FM  45.9 mg/100 g FM  59.9 mg/100 g FM  50.9 mg/100 g FM | (Cendrowski et al., 2017) |
| *Rosa rugosa* petals | Anthocyanins | Cyanidin 3,5-*O*-diglucoside  Peonidin 3-*O*-sophoroside  Peonidin 3,5-*O*-diglucoside  Peonidin 3-*O*-diglucoside | 27.4 mg/100 g FM  1.62 mg/100 g FM  149.1 mg/100 g FM  2.4 mg/100 g FM | (Cendrowski et al., 2017) |
| *Rosa damascena* petals | Phenolics | Total phenolic | 212.1 mg GAE/g | (Trendafilova et al., 2023) |
| Roselle calyx | Anthocyanin | Cyanidin-3-*O*-glucoside  Cyanidin 3-xylosylglucoside  Delphinidin-3-sambubioside  Cyanidin-3-sambubioside. | 40.5 µg/g FM 150 mg/100 g FM  955.1 µg/g FM  531.4 µg/g FM | (Lema et al., 2021; Li et al., 2022) |
| Roselle calyx | Phenolic acid | protocatechuic acid | 2.1 mg/g DM | (Osman et al., 2014) |
| Roselle calyx | Phenolic acid | chlorogenic acid | 1.0 mg/g DM | (Samadi & Raouf Fard, 2020) |
| Roselle calyx | flavonoid | quercetin | 0.2 mg/g DM | (Samadi & Raouf Fard, 2020) |
| Roselle calyx | Phenolics | Total phenolic | 38.5 mg GAE/g | (Riaz et al., 2020) |

DM: Dry matter; FM: Fresh matter

**Table S4**. Minerals in rose and roselle with their daily recommended intake for adult.

| **Mineral**  **(recommended adult intake)** | **Amount** | **Function** | **Reference** |
| --- | --- | --- | --- |
| ***Rosa damascena* fruit flesh and seed** | | | |
| Potassium  (3510 mg/day) | 12454 mg/kg FM  2243 mg/kg FM | Supports nerve function and maintains fluid balance. | (Kazaz et al., 2009; World Health Organization, 2012) |
|  |  |  |  |
| Calcium  (1000-1200 mg/day) | 11162 mg/kg FM  3885 mg/kg FM | Supports bone health and muscle function. | (Kazaz et al., 2009; National Institutes of Health Office of Dietary Supplements, 2025a) |
| Magnesium  (310-420 mg/day) | 1501 mg/kg FM  441 mg/kg FM | Important for enzyme activity and regulating blood sugar. | (Kazaz et al., 2009; National Institutes of Health Office of Dietary Supplements, 2025c) |
| Iron  (8-27 mg/day) | 118 mg/kg FM  110 mg/kg FM | Important for oxygen transport in the blood. | (Kazaz et al., 2009; National Institutes of Health Office of Dietary Supplements, 2025b) |
| Phosphorus  (700 mg/day) | 1459 mg/kg FM  720 mg/kg FM | Important for energy production and cellular function. | (Kazaz et al., 2009; National Institutes of Health Office of Dietary Supplements, 2025e) |
| Zinc  (8-11 mg/day) | 13 mg/kg FM  10 mg/kg FM | Helps in skin regeneration and wound closure | (Kazaz et al., 2009; National Institutes of Health Office of Dietary Supplements, 2025f) |
| Manganese  (2.3 mg/day) | 73 mg/kg FM  24 mg/kg FM | Protect cells from oxidative damage and support bone formation | (Kazaz et al., 2009; National Institutes of Health Office of Dietary Supplements, 2025d) |
| **Roselle seed and calyx** | | | |
| Calcium  (1000 mg/day) | 869 mg/100g  1675 mg/100g, 2105 mg/100g DM | Promotes bone health, muscle function | (Ajayi & Oyerinde, 2020; National Institutes of Health, Office of Dietary Supplements, 2025a; Riaz et al., 2020; Salami & Afolayan, 2021) |
| Magnesium  (310-420 mg/day) | 278 mg/100g  440 mg/100g,  280 mg/100g  DM | Supports energy production and protein synthesis. | (Ajayi & Oyerinde, 2020; National Institutes of Health, Office of Dietary Supplements, 2025c; Riaz et al., 2020; Salami & Afolayan, 2021) |
| Phosphorus  (700 mg/day) | 125 mg/100 g | Important for energy production and cellular function. | (National Institutes of Health, Office of Dietary Supplements, 2025e; Salami & Afolayan, 2021) |
| Potassium  (3510 mg/day) | 20.3 mg/100g  2265 mg/100g,  1263 mg/100 g DM | Supports nerve function, maintains fluid balance | (Ajayi & Oyerinde, 2020; Riaz et al., 2020; Salami & Afolayan, 2021; World health organization, 2012) |
| Iron  (8-27 mg/day) | 3.7 mg/100g  21 mg/100g  DM | Important for oxygen transport in the blood. | (Ajayi & Oyerinde, 2020; National Institutes of Health, Office of Dietary Supplements, 2025; Riaz et al., 2020; Salami & Afolayan, 2021) |
| Zinc  (8-11 mg/day) | 4 mg/100 g,  6 mg/100 g DM | Helps in skin regeneration and wound closure. | (National Institutes of Health, Office of Dietary Supplements, 2025; Riaz et al., 2020; Salami & Afolayan, 2021) |
| Manganese  (2.3 mg/day) | 29 mg/100 g DM | Protects cells from oxidative damage and support bone formation | (National Institutes of Health, Office of Dietary Supplements, 2025; Salami & Afolayan, 2021) |

DM: Dry matter; FM: Fresh matter

**Table S5**. Organic acids concentration in rose and in roselle

| **Plant part** | **Citric acid** | **Oxalic acid** | **Malic acid** | **Quinic acid** | **Tartaric acid** | **Ascorbic acid** | **Reference** |
| --- | --- | --- | --- | --- | --- | --- | --- |
| Rose petals | 1.2 g/100g DM | 0.3 g/100g DM | 1.2 g/100g DM | 1.5 g/100g DM | N/A | N/A | (Pires et al., 2017) |
| *Rosa* spp. | 0.7 mg/g FM | N/A | 6.1 mg/g FM | N/A | N/A | 0.6 mg/g FM | (Vukosavljev et al., 2023) |
| Roselle calyces | 12-20% | 44% | 9-47% | N/A | 3-8% | 0-3% | (Al-Wandawi, 2015; Izquierdo-Vega et al., 2020) |
| Roselle green leaves | 0% | 74% | 22% | N/A | 2% | 1% | (Al-Wandawi, 2015) |
| Roselle petioles | 1% | 87% | 11% | N/A | 1% | 1% | (Al-Wandawi, 2015) |
| Roselle seed pods | 73% | 19% | 2% | N/A | 4% | 2% | (Al-Wandawi, 2015) |

DM: Dry matter; FM: Fresh matter Rose values are reported on a dry-matter (DM) or fresh-matter (FM) mass basis; roselle values are reported as a percentage of total organic acid content, as given in the original sources.

**Table S6**: Products developed from rose and roselle plants

| Plant/ Plant part | Products | Observation | Reference |
| --- | --- | --- | --- |
| Rose powder (*Rosa damascena*) | Cookies | 7.5% inclusion of rose powder was acceptable during sensory; 10% inclusion level yielded 168 g/kg GAE TPC. | (Gül & Tekeli̇, 2019) |
| Rose petal powder | Cookies | 5% inclusion of rose powder scored best for the sensory quality, and lower thickness in all cookies incorporated with rose powder (3%, 5%, and 10%) ranged between 9.9 - 10.9 mm, compared to the control cookies, 11.05 mm. | (Hnin et al., 2021) |
| Rose dry petals (*Rosa rugosa*) | Liqueur | 70% ethanol used, total phenols in the conventional process were 93 mg/g, maceration process 76 mg/g in white dry *Rosa rugosa* petals. Total anthocyanins were 12 mg/g and 15 mg/g in conventional and macerated dry petals, respectively. | (Fascella et al., 2022) |
| Roselle powder | Tea | 100% roselle powder yielded 41 mg/GAE/g, 4 mg/100g iron, 869 mg/100g calcium, 20 mg/100g potassium, and 278 mg/100g magnesium. | (Ajayi & Oyerinde, 2020) |
| Rose petals | Rose oil | Β‑citronellol range was 37 -39% in damask rose, 38% in kushui rose, and 48% in *Rosa rugosa*. | (Dobreva & Nedeltcheva-Antonova, 2023) |
| Rose petals | Rose water | Concentration in cosmetics is 0-33%, rose water is formed from 2 ml rose oil, 2 ml ethanol (90%), and 100 ml purified water. | (Mahboubi & Lu, 2024) |
|  |  |  |  |
| Rose flower petals | Rose vinegar | Alcohol is used for the first stage at 25° C for 30 days, and acetic acid is used in the second stage of vinegar fermentation at 25° C for 30 days. Rose water was inoculated with Saccharomyces cerevisiae (10^6^ log CFU/mL) | (Özdemir & Budak, 2022) |
| Roselle flower petals | Extract, muffins | Extract volume 45 mL, citric acid 1.1 g, sodium bicarbonate 1.7 g, anthocyanins 127 mg/100 g, phenolic 13 mg/100g, 84% sensory score. | (Marak et al., 2022) |
| Roselle stems | Spice mix | 7 g roselle stems powder + 21g tamarind powder + 2g Salt + 4g onion powder + 4g tomato powder + 2g taro powder + 2g monosodium glutamate (MSG) + 2g sugar. Roselle powder as a souring agent in a spice mix was acceptable. | (Ibrahim et al., 2023) |
| Rose petals | Rose jam | 250 g rose petals, 54-55 g fresh ginger root, and 88-90 g fresh lemon. | (Rădulescu et al., 2021) |

GAE: Gallic Acid Equivalent TPC: Total Phenolic Content; CFU: Colony Forming Unit

Ajayi, O. A., & Oyerinde, M. O. (2020). Evaluation of Nutritional composition of Roselle (*Hibiscus sabdariffa*) herbal Tea infused with Ginger (*Zingiber officinale*) and Lemon (Citrus limon) Peel. Australian Journal of Science and Technology, 4(1), 215–221.

Al-Wandawi, H. (2015). Organic acids composition of different parts of the medicinal plant/roselle (*Hibiscus sabddariffa*). International Journal of Biological & Pharmaceutical Research, 6(10), 808-813.

Cendrowski, Andrzej, Ścibisz, I., Mitek, M., Kieliszek, M., & Kolniak-Ostek, J. (2017). Profile of the Phenolic Compounds of *Rosa rugosa* Petals. Journal of Food Quality, 2017, 1–10. https://doi.org/10.1155/2017/7941347

de Lima Franzen, F., Rodrigues de Oliveira, M. S., Lidório, H. F., Farias Menegaes, J., & Martins Fries, L. L. (2019). Chemical composition of rose, sunflower and calendula flower petals for use in human food. Agricultural Science and Technology, 20 (1), 149-168, 20(1). https://doi.org/https://doi.org/10.21930/rcta.vol20_num1_art:1252

Dobreva, A., & Nedeltcheva-Antonova, D. (2023). Comparative Chemical Profiling and Citronellol Enantiomers Distribution of Industrial-Type Rose Oils Produced in China. Molecules, 28(3), 1281. https://doi.org/10.3390/molecules28031281

El-Deab, S. M., & Ghamry, H. E. (2017). Nutritional Evaluation of Roselle Seeds Oil and Production of Mayonnaise. Int J. Food Sci. Nutr. Eng 7, No. 2 (2017): 32-37.

Fascella, G., D’Angiolillo, F., Mammano, M. M., Granata, G., & Napoli, E. (2022). Effect of Petal Color, Water Status, and Extraction Method on Qualitative Characteristics of Rosa rugosa Liqueur. Plants, 11(14), 1859. https://doi.org/10.3390/plants11141859

Ghislain, M. T., Etengeneng, A. E., Sonia, M. N. H., Noelle, K. S. C., & Innocent, G. (2014). Proximate and Mineral Composition, Protein Quality of Hibiscus Sabdariffa L. (Roselle) Seeds Cultivated in Two Agro Ecological Areas in Cameroon. International Journal of Nutrition and Food Sciences, 3(4), 251. https://doi.org/10.11648/j.ijnfs.20140304.14

Gül, H., & Tekeli̇, S. G. (2019). Inclusion of *Rosa damascena* Mill. Powder into cookies: Nutritional, antioxidant and quality characteristics. International Journal of Agriculture Forestry and Life Sciences, 3(2), 301-306.

Hainida, K. I. E., Amin, I., Normah, H., & Mohd.-Esa, N. (2008). Nutritional and amino acid contents of differently treated Roselle (*Hibiscus sabdariffa* L.) seeds. Food Chemistry, 111(4), 906–911. https://doi.org/10.1016/j.foodchem.2008.04.070

Hegde, A. S., Gupta, S., Sharma, S., Srivatsan, V., & Kumari, P. (2022). Edible rose flowers: A doorway to gastronomic and nutraceutical research. Food Research International, 162, 111977. https://doi.org/10.1016/j.foodres.2022.111977

Hnin, K. K., Zhang, M., & Wang, B. (2021). Development of nutritional properties in cookies with the incorporation of different levels of rose flower powder by microwave-vacuum drying. Drying Technology, 39(9), 1136–1148. https://doi.org/10.1080/07373937.2020.1725035

Ibrahim, A. M., Blas, R. D. M., Batobato, M. L. A., Norcos, C. J. A., Miranda, J. C. S., Sevilla, F. G., & Baron, D. L. O. (2023). Sensory Acceptability of Roselle Leaves (*Hibiscus sabdariffa*) as Souring Agent in Instant Spice Mix. Journal of Tourism, Hospitality & Culinary Arts, 15(1), 78-88.

Izquierdo-Vega, J., Arteaga-Badillo, D., Sánchez-Gutiérrez, M., Morales-González, J., Vargas-Mendoza, N., Gómez-Aldapa, C., Castro-Rosas, J., Delgado-Olivares, L., Madrigal-Bujaidar, E., & Madrigal-Santillán, E. (2020). Organic Acids from Roselle (*Hibiscus sabdariffa* L.). A Brief Review of Its Pharmacological Effects. Biomedicines, 8(5), 100. https://doi.org/10.3390/biomedicines8050100

Kazaz, S., Baydar, H., & Erbas, S. (2009). Variations in chemical compositions of *Rosa damascena* Mill. And *Rosa canina* L. fruits. Czech Journal of Food Sciences, 27(3), 178–184. https://doi.org/10.17221/5/2009-CJFS

Kholaf, G. M. (2024). The Colour Stability and Physico-Chemical Properties of Tomato Paste Enriched with Rosella (*Hibiscus sabdariffa* L.). Ournal of Food and Dairy Sciences, 15(11), 159-166., 15.

Kumari, P., Ujala, & Bhargava, B. (2021). Phytochemicals from edible flowers: Opening a new arena for healthy lifestyle. Journal of Functional Foods, 78, 104375. https://doi.org/10.1016/j.jff.2021.104375

Lee, J. H., Lee, H.-J., & Choung, M.-G. (2011). Anthocyanin compositions and biological activities from the red petals of Korean edible rose (*Rosa hybrida* cv. *Noblered*). Food Chemistry, 129(2), 272–278. https://doi.org/10.1016/j.foodchem.2011.04.040

Lema, A. A., Mahmod, N. H., Khandaker, M. M., & Abdulrahman, M. D. (2021). Roselle anthocyanin stability profile and its potential role in post-harvest deterioration: A review. Plant Science Today. https://doi.org/10.14719/pst.1336

Li, J., Li, Y., Li, M., Lin, L., Qi, J., Xu, J., Zhang, L., Fang, P., & Tao, A. (2022). Novel Insights into Anthocyanin Synthesis in the Calyx of Roselle Using Integrated Transcriptomic and Metabolomic Analyses. International Journal of Molecular Sciences, 23(22), 13908. https://doi.org/10.3390/ijms232213908

Liu, Y., Hip Seng, Y., Hock Eng Khoo, K., & Vui Fui, V. (2024). Nutritional Properties of Flower Petals of Four *Rosa Hybrida* Cultivars. SciBase Human Nutrition and Food Science, 1(2). https://doi.org/10.52768/SciBaseHumNutrFoodSci/1007

Mahboubi, M., & Lu, S. H. (2024). Rose water, quality control, and its application. Journal of Medicinal Plants, 23(91), 1–15. https://doi.org/10.61186/jmp.23.91.1

Mahmoud, R., Ghanem, H., Wahed, N., & Ateya, A. (2024). Assessing the Viability of Incorporating Sunflower Meal and Roselle Seed Meal as Unconventional Protein Sources in the Diets of Japanese Quails on Growth Performance, Carcass Characteristics, Serum Metabolites, Gene Expression, and Economic Efficienc. Egyptian Journal of Veterinary Sciences, 55(5), 1371–1386. https://doi.org/10.21608/ejvs.2024.248294.1670

Marak, S., Kaushik, N., Dikiy, A., Shumilina, E., & Falch, E. (2022). Nutritionally Enriched Muffins from Roselle Calyx Extract Using Response Surface Methodology. Foods, 11(24), 3982. https://doi.org/10.3390/foods11243982

Mlcek, J., Plaskova, A., Jurikova, T., Sochor, J., Baron, M., & Ercisli, S. (2021). Chemical, Nutritional and Sensory Characteristics of Six Ornamental Edible Flowers Species. Foods, 10(9), 2053. https://doi.org/10.3390/foods10092053

National Institutes of Health Office of Dietary Supplements. (2025a). Calcium Fact Sheet for Health Professionals [Grey report]. NIH Office of Dietary Supplements. https://ods.od.nih.gov/factsheets/Calcium-HealthProfessional/

National Institutes of Health Office of Dietary Supplements. (2025b). Iron Fact Sheet for Health Professionals [Grey report]. NIH Office of Dietary Supplements. https://ods.od.nih.gov/factsheets/Iron-HealthProfessional/

National Institutes of Health Office of Dietary Supplements. (2025c). Magnesium Fact Sheet for Health Professionals [Grey report]. NIH Office of Dietary Supplements. https://ods.od.nih.gov/factsheets/Magnesium-Consumer/

National Institutes of Health Office of Dietary Supplements. (2025d). Manganese Fact Sheet for Health Professionals [Grey report]. NIH Office of Dietary Supplements. https://ods.od.nih.gov/factsheets/Manganese-HealthProfessional/

National Institutes of Health Office of Dietary Supplements. (2025e). Phosphorus Fact Sheet for Health Professionals [Grey report]. NIH Office Dietary Supplements. https://ods.od.nih.gov/factsheets/Phosphorus-HealthProfessional/

National Institutes of Health Office of Dietary Supplements. (2025f). Zinc Fact Sheet for Health Professionals [Grey report]. NIH Office Dietary Supplements. https://ods.od.nih.gov/factsheets/Zinc-HealthProfessional/

Osman, M., Sazalli, N. N. H., Willium, D., Golam, F., & Nezhadahmadi, A. (2014). Potentiality of Roselle and Onion (*Allium cepa*) peel as Raw Materials for Producing Protocatechuic Acid in Tropical Malaysia: A Comparative Study. Indian Journal of Science and Technology, 7(11), 1847–1851. https://doi.org/10.17485/ijst/2014/v7i11.6

Özdemir, N., & Budak, N. H. (2022). Bioactive compounds and volatile aroma compounds in rose Rosa damascena Mill vinegar during the aging period. Food Bioscience, 50, 102062. https://doi.org/10.1016/j.fbio.2022.102062

Pires, T. C. S. P., Dias, M. I., Barros, L., & Ferreira, I. C. F. R. (2017). Nutritional and chemical characterization of edible petals and corresponding infusions: Valorization as new food ingredients. Food Chemistry, 220, 337–343. https://doi.org/10.1016/j.foodchem.2016.10.026

Rădulescu, L., Bordean, D.-M., Hădărugă, N. G., Megyesi, C. I., & Rinovetz, A. E. (2021). Nutritional data evaluation study on rose petals, ginger root and lemon. Journal of Agroalimentary Processes and Technologies.

Riaz, G., Naik, S. N., Garg, M., & Chopra, R. (2020). Phytochemical Composition of an Underutilized Plant Sorrel/Roselle (*Hibiscus Sabdariffa* L.) Cultivated in India. Letters in Applied NanoBioScience, 10(2), 2138–2147. https://doi.org/10.33263/LIANBS102.21382147

Salami, S. O., & Afolayan, A. J. (2021). Evaluation of nutritional and elemental compositions of green and red cultivars of roselle: *Hibiscus sabdariffa* L. Scientific Reports, 11(1), 1030. https://doi.org/10.1038/s41598-020-80433-8

Samadi, S., & Raouf Fard, F. (2020). Phytochemical properties, antioxidant activity and mineral content (Fe, Zn and Cu) in Iranian produced black tea, green tea and roselle calyces. Biocatalysis and Agricultural Biotechnology, 23, 101472. https://doi.org/10.1016/j.bcab.2019.101472

Shameh, S., Hosseini, B., Alirezalu, A., & Maleki, R. (2018). Phytochemical Composition and Antioxidant Activity of Petals of Six Rosa Species from Iran. Journal of AOAC INTERNATIONAL, 101(6), 1788–1793. https://doi.org/10.5740/jaoacint.18-0111

Trendafilova, A., Staleva, P., Petkova, Z., Ivanova, V., Evstatieva, Y., Nikolova, D., Rasheva, I., Atanasov, N., Topouzova-Hristova, T., Veleva, R., Moskova-Doumanova, V., Dimitrov, V., & Simova, S. (2023). Phytochemical Profile, Antioxidant Potential, Antimicrobial Activity, and Cytotoxicity of Dry Extract from *Rosa damascena* Mill. Molecules, 28(22), 7666. https://doi.org/10.3390/molecules28227666

Vukosavljev, M., Stranjanac, I., Van Dongen, B. W. P., Voorrips, R. E., Miric, M., Bozanic Tanjga, B., Arens, P., & Smulders, M. J. M. (2023). A novel source of food – garden rose petals. Acta Horticulturae, (1362), 165–172. https://doi.org/10.17660/ActaHortic.2023.1362.22

World Health Organization (Ed.). (2012). Guideline: Potassium Intake for Adults and Children. World health organization. https://www.who.int/publications/i/item/9789241504829

World health organization (Ed.). (2012). Potassium intake for adults and children: Guideline. World health organization.
